# Compatibility of Drotaverine Hydrochloride with Ibuprofen and Ketoprofen Nonsteroidal Anti-Inflammatory Drugs Mixtures

**DOI:** 10.3390/ma15031244

**Published:** 2022-02-08

**Authors:** Andreia-Cristina Soare, Viorica Meltzer, Claudiu Colbea, Ioana Stanculescu, Elena Pincu

**Affiliations:** Department of Physical Chemistry, Faculty of Chemistry, University of Bucharest, Bd. Regina Elisabeta 4-12, 030018 Bucharest, Romania; andreia-cristina.soare@drd.unibuc.ro (A.-C.S.); meltzerviorica@yahoo.com (V.M.); ccolbea@ethz.ch (C.C.); ioana.stanculescu@chimie.unibuc.ro (I.S.)

**Keywords:** DSC, eutectic, NSAIDs, Drotaverine Hydrochloride, excess functions

## Abstract

Formulations with two or more active pharmaceutical ingredients (APIs) are a researched trend due to their convenient use compared with multiple medications. Moreover, drug-drug combinations may have a synergistic effect. Drotaverine hydrochloride (D-HCl) is commonly used for its antispasmodic action. The combination of a spasmolytic and an analgesic drug such as ibuprofen (Ibu) or ketoprofen (Ket) could become the answer for the treatment of localized pain. D-HCl:Ibu and D-HCl:Ket drug-drug interactions leading to the formation of eutectic compositions with increased bioavailability, obtained by mechanosynthesis, a green, solvent-free method was explored for the first time. The compatibility of Ibuprofen, Ketoprofen, and Drotaverine Hydrochloride was investigated using differential scanning calorimetry (DSC), X-ray diffraction (XRD), and Fourier-Transform Infrared spectroscopy (FTIR). Solid-liquid equilibrium (SLE) phase diagrams for the binary systems of active pharmaceutical ingredients were developed and the Tammann diagrams were designed to determine the eutectic compositions. The excess thermodynamic functions *G^E^* for the pre-, post-, and eutectic compositions were obtained using the computed activity coefficients data. Results show that drotaverine-based pharmaceutical forms for pain treatment may be obtained at 0.9 respectively 0.8 molar fractions of ibuprofen and ketoprofen which is advantageous because the maximum allowed daily dose of Ibu is about 6 times higher than those of D-HCl and Ket. The obtained eutectics may be a viable option for the treatment of pain associated with cancer therapy.

## 1. Introduction

Nowadays, multiple medications are an issue that the pharmaceutical industry tries to address by developing new formulations containing two or more active pharmaceutical ingredients (APIs) that act simultaneously and synergistically, eventually without having adverse effects on the body. An optimal formulation of this kind can be achieved by forming cocrystals or eutectic mixtures [1,2]. Pharmaceutical cocrystals may potentiate the physicochemical and mechanical properties of the substances [3], bioavailability [4], solubility [4,5] or stability [3,6] as well as in vivo activity [1], while a eutectic mixture behaves like a single pure substance having a melting temperature lower than that of the components which is correlated with increased bioavailability [7]. Usually, cocrystals are composed of an API and another substance whose role is to potentiate its properties called coformer, and are obtained by different techniques: (i) traditional, solvent evaporation, solvent reduced technique, or mechanochemical processing-solid state grinding, and (ii) advanced: microwave-assisted synthesis or supercritical fluid technology [2,8]. Nowadays, in the pharmaceutical industry more and more often this coformer is another API.

**Figure 1:**
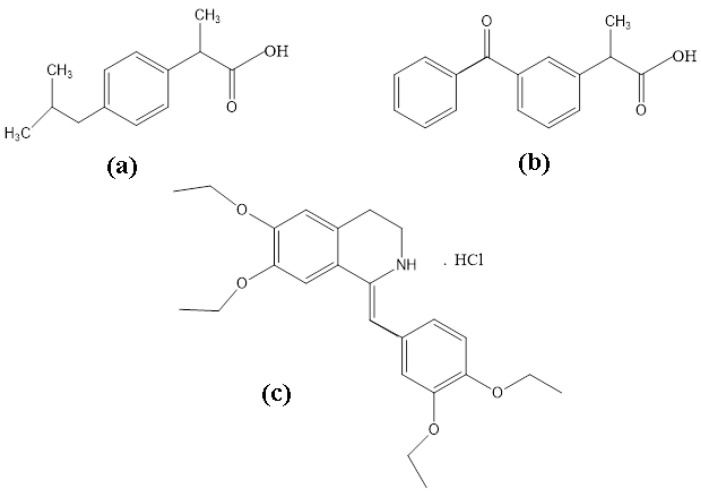
Structural formula for: (**a**) Ibuprofen (Ibu), (**b**) Ketoprofen (Ket) and (**c**) Drotaverine Hydrochloride (D-HCl).

Ibuprofen (RS)-2-(4-(2-methylpropyl) phenyl) propionic acid (Figure 1a) and Ketoprofen (RS) 2-(3-benzophenyl)-propionic acid (Figure 1b) are propionic acid derivatives with analgesic, anti-inflammatory and antipyretic actions [9]. They belong to the class of non-steroidal anti-inflammatory drugs (NSAIDs), work by inhibiting the body’s production of prostaglandin, and are competitive inhibitors of cyclooxygenase (COX1 and COX2) [10]. Drotaverine Hydrochloride 1-(3, 4-diethoxybenzylidene)-6,7-methoxy-1,2,3,4-tetrahydroisoquinoline hydrochloride (Figure 1c) is an antispasmodic agent structurally related to papaverine [11,12], being a selective inhibitor of phosphodiesterase 4, with no anticholinergic effects. Recent studies on the action of this active substance on human cancer cell lines show that drotaverine can be used as a cytostatic agent, in addition to its normal use [13].

The source of pain is usually the muscle spasm. Antispasmodic and analgesic drugs are used to remove this source. Among the most commonly used active pharmaceutical ingredients with analgesic action is Ibuprofen (Ibu), while Drotaverine Hydrochloride (D-HCl) is employed for the antispasmodic effect. A binary system of active pharmaceutical substances with antispasmodic and analgesic action is Drotaverine Hydrochloride and Mefenamic acid mixture that is indicated for the symptomatic treatment of spasmodic dysmenorrhea, colic pains (including ureteric), biliary and intestinal colic [11,12].

The objective of this study was to obtain and characterize new binary mixtures between drotaverine hydrochloride and non-steroidal anti-inflammatory drugs (NSAIDs) Ibuprofen and Ketoprofen respectively, not reported in the literature. The behavior of these binary mixtures was determined from the phase diagrams and the Tammann plots based on the DSC experiments. The excess thermodynamic function *G^E^* for the pre-, post-, and eutectic composition was obtained using the computed activity coefficients data. For the first time, the compatibility of Ibuprofen, Ketoprofen, and Drotaverine Hydrochloride was investigated using differential scanning calorimetry (DSC), X-ray diffraction (XRD), and Fourier-Transform Infrared spectroscopy (FTIR).

The novelty of this study is that binary mixtures of drotaverine hydrochloride and NSAIDS (Ibu and Ket), one with antispasmodic action and another with analgesic one, could be the solution against localized pain and in the treatment of cancer. These combinations can be studied on several human tumor cell lines [10,13].

## 2. Materials and Methods

### 2.1. Chemicals

Commercially available Ibuprofen, C_13_H_18_O_2_ with molecular mass 206.29 g mol^−1^ (white powder, *p* ≥ 0.98, Sigma-Aldrich, Schnelldorf, Germany), Drotaverine Hydrochloride, C_24_H_31_NO_4_ HCl with molecular mass 433.97 g mol^−1^ (yellow powder, *p* ≥ 0.98, Ra Chem Pharma Ltd., Hyderabad, India), Ketoprofen, C_16_H_14_O_3_ with molecular mass 254.281 g mol^−1^ (white powder, *p* ≥ 0.98, Società Italiana Medicinali Scandicci, Filarone, Italy) were used without further purification.

### 2.2. Equipment

Thermal analysis measurements were performed with a Perkin Elmer Diamond DSC device (Waltham, MA, USA) with a heating/cooling rate of 1 K min^−1^ on a temperature interval between 293 K and 550 K. The measurements were made in an inert gas atmosphere, argon, at a flow rate of 20 mL min^−1^. The instrument has been calibrated for specific parameters, temperature, and enthalpy using high purity indium (Δ*_fus_H* = 28.54 J g^−1^). All samples’ masses were between 7 and 10 mg and were weighed with the Partner XA balance (Radwag, Radom, Poland) with a precision of 10 μg.

The XRD measurements for the crystalline structure of the samples were studied by X-Ray Diffraction using a D8 Advance Bruker diffractometer (Cu Kα radiation λ = 1.5418 Å, 40 kV, 40 mA, Bragg-Bretano geometry, Karlsruhe, Germany) at a scanning speed of 0.10 degrees/min in the 10–40 degrees 2Θ range. Crystallite size was estimated with Scherrer equation.

All the FTIR measurements were performed on a Perkin Elmer Spectrum Two ATR-FTIR (Waltham, MA, USA) with a data acquisition count set to 100. The spectrometer was equipped with an universal attenuated total reflection (UATR) accessory containing a diamond/ZnSe crystal for 1 reflection analysis. FT-IR spectra were recorded at a 4 cm^−1^ spectral resolution.

### 2.3. Preparation of the Binary Mixtures

Preparation of physical mixtures was carried out by weighing different ratios of API and mixing them using a mortar and pestle. Various molar fractions 0, 0.2, 0.4, 0.5, 0.7, 0.9, 1 of the binary mixtures of APIs: Drotaverine Hydrochloride—Ibuprofen and Drotaverine Hydrochloride—Ketoprofen were made for the entire concentration interval, 0.00–1.00, by weighing, milling and homogenization at room temperature.

### 2.4. Data Analysis

The enthalpy was calculated by integrating the area under the peak using the DSC software, Pyris. OriginPro 6.0 (Northampton, MA, USA) was employed for the data treatment after ASCII conversion.

Determination of the solid-liquid equilibrium phase diagram for a mixture in which its components are not miscible in the solid phase but exhibits an ideal behavior in the liquid phase, *γ_i_* = 1 was performed using the Schroeder van Laar equation [7,14]:

(1) $$lnx_{i}=-\left(\Delta_{fus}H_{i}^{o}/R\right)\left(1/T-1/T_{fus,i}\right)$$

where *x_i_* is the mole fraction of the components at the temperature *T*, *R* is the gas constant, $\Delta_{fus}H_{i}^{o}$ is the molar enthalpy of fusion of component *i* (*i* = 1, 2), and *T_fus,i_* is the melting temperature of the pure component.

Using Equation (1) one can determine the melting temperatures corresponding to mixtures of various compositions with the relation:

(2) $$T=-(\Delta_{fus}H_{i}^{o}/R)/(lnx_{i}-\Delta_{fus}H_{i}^{o}/RT_{i}^{o})$$

With this temperature, the ideal phase diagram was created.

To characterize the eutectic fluid and specify its structure we use the value of the mixing enthalpy [15,16], which represents the difference between the melting enthalpy of the system with real behavior and the melting enthalpy calculated additively if it is considered that the eutectic is a simple mechanical mixture of components which does not involve any association in the melt.

(3) $$\Delta^{M}H={\left(\Delta_{fus}H^{o}\right)}_{exp}-{\left(\Delta_{fus}H^{o}\right)}_{calc}$$

where ${\left(\Delta_{fus}H^{o}\right)}_{exp}$ represents the molar enthalpy of fusion determined from DSC experiments and ${\left(\Delta_{fus}H^{o}\right)}_{calc}$ represents the corresponding calculated value using the relationship:

(4) $${\left(\Delta_{fus}H^{o}\right)}_{calc}=x_{1}\Delta_{fus}H_{1}^{o}+x_{2}\Delta_{fus}H_{2}^{o}$$

Three types of structures were proposed depending on the sign and size of the mixing enthalpy [15]: quasi-eutectic for which Δ*^M^H* > 0, clustering of molecules when Δ*^M^H* < 0, and molecular solutions when Δ*^M^H* = 0.

To characterize the deviation from the ideal behavior of the systems and also to have an overview of the nature of interactions between the components forming the eutectic we calculated the excess Gibbs function with the following relation [17]:

(5) $$G^{E}=RT\left(x_{1}ln\gamma_{1}^{1}+x_{2\;}ln\gamma_{2}^{1}\right)$$

where *x_i_*, $\gamma_{i}^{i}$ are the mole fraction and the activity coefficient in the liquid state of component *i*.

The activity coefficient of a component *i* present in the eutectic melt, neglecting the difference in heat capacity of the liquid and solid phases, can be calculated from equation [18]:

(6) $$ln\left(x_{i}\gamma_{i}\right)=-(\Delta_{fus}H_{i}^{o}/R)(1/T-1/T_{fus,i\;})$$

where $x_{i}^{l}$, $\gamma_{i}^{l}$, $\Delta_{fus}H_{i}^{o}$ and *T_fus_*_,*i*_ are the mole fraction, activity coefficient, heat of fusion, and the melting temperature of component *i*, *R* is the gas constant, and *T* is the liquidus temperature.

## 3. Results and Discussion

### 3.1. DSC Analysis of Pure Substances

**Figure 2:**
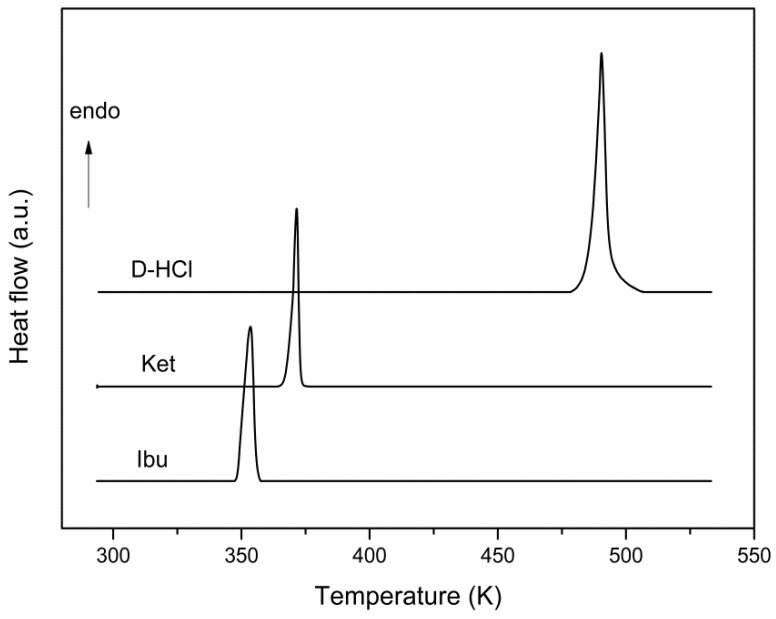
DSC curves for Ibuprofen (Ibu), Ketoprofen (Ket) and Drotaverine Hydrochloride (D-HCl).

In order to obtain the APIs solid-liquid phase diagrams, the DSC characterization was performed. The corresponding DSC curves (Figure 2) show only the melting processes of APIs: Ibuprofen at 348.4 ± 0.2 K, Ketoprofen at 367.5 ± 0.2 K, and Drotaverine Hydrochloride at 488.4 ± 0.4 K. These experimental data are in accordance with literature data [18,19,20].

The thermodynamic parameters of the melting process shown in the DSC curves are presented in Table 1.

### 3.2. DSC Analysis of Binary Mixtures of API

The DSC curves corresponding to binary mixtures of API have two endothermic peaks: the first is associated with the melting process of the eutectic mixture and the second is associated with the melting of the excess component.

**Figure 3:**
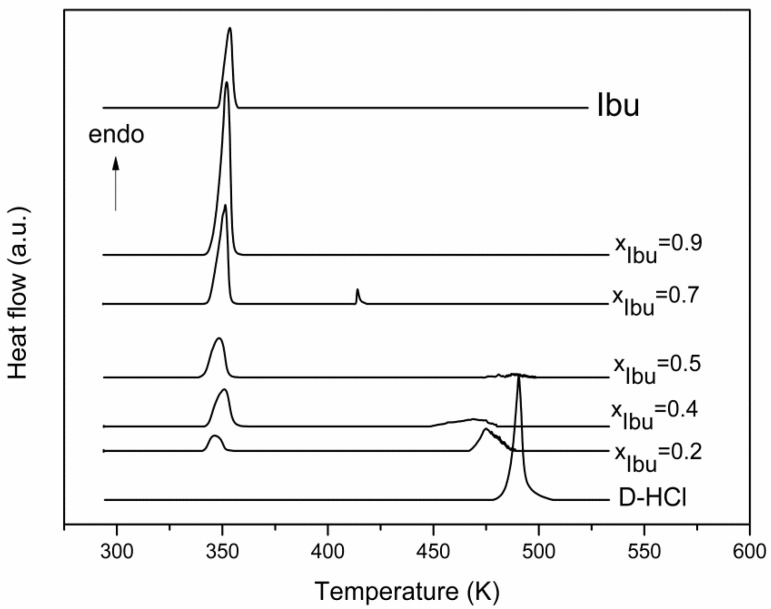
DSC curves for Ibu and D-HCl and their binary mixtures.

For the binary mixture of Ibuprofen and Drotaverine Hydrochloride, the DSC curves of different compositions are presented in Figure 3.

For the Ibu—D-HCl mixture, the eutectic mixture corresponding to a molar fraction of 0.9 Ibu is characterized by a melting point of 343.8 K with a higher melting enthalpy of eutectic composition, Δ*_fus_H* = 111.9 J g^−1^.

The thermodynamic parameters of the melting process shown in the DSC curves are presented in Table 2.

**Figure 4:**
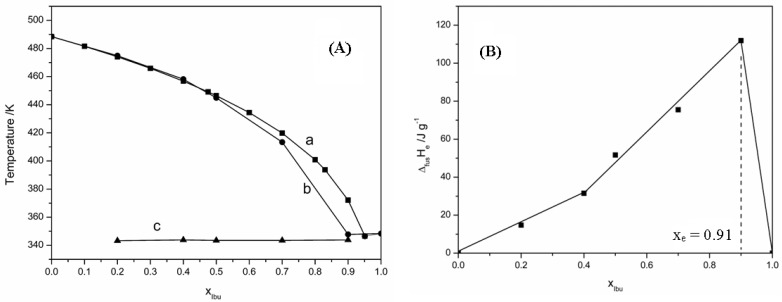
(**A**). Phase diagram of binary mixture Ibu—D-HCl: a. ideal behavior; b. real behavior; c. eutectic temperature. (**B**). Tammann’s plot.

The phase diagram calculated using the ideal Schroder van Laar equation and the real phase diagram obtained using the DSC data is presented in Figure 4A as a temperature-composition plot.

For precise determination of eutectic composition, the melting enthalpy was plotted according to the molar fraction of Ibuprofen, and the Tammann diagram shown in Figure 4B was obtained.

According to the Tammann diagram, the eutectic composition corresponds to a molar fraction of 0.91 Ibu. As expected, the enthalpy value of the eutectic reaction increased linearly until the exact composition of the eutectic point and then decreased linearly until crossing the composition axis at its extremities. The phase diagram shows the ideal behavior of the Ibu—D-HCl mixture in the range of 0 to 0.4 molar fraction of Ibuprofen and a deviation from ideal behavior at higher molar fractions. The same behavior is observed in Tammann’s plot.

The analysis of the excess Gibbs free energy values indicates that they are positive on the molar fractions range 0–0.8 which shows that between molecules of the same type occur strong interactions, while at the eutectic point the value of *G^E^* is negative and shows the association between the molecules of Ibuprofen and Drotaverine Hydrochloride.

The formation of clusters of molecules is also reflected by the value of the mixing enthalpy that was calculated using relation (3) and which is equal to −3.09 kJ mol^−1^.

**Figure 5:**
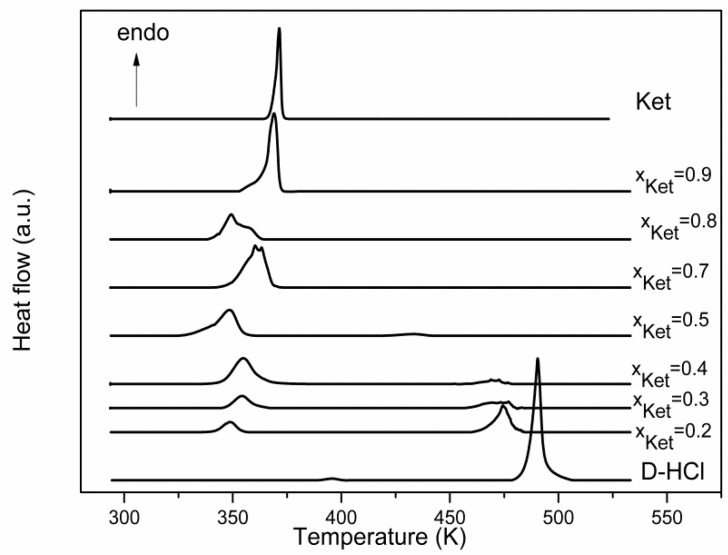
DSC curves for Ket and D-HCl and their binary mixtures.

The binary mixture of different compositions of Ketoprofen and Drotaverine Hydrochloride was characterized by DSC, the curves obtained are presented in Figure 5.

The eutectic composition of binary mixture Ket—D-HCl corresponds to a molar fraction of 0.8 Ket, having a melting temperature of 344.1 K and a melting enthalpy of 94.02 J g^−1^.

The thermodynamic parameters of the melting process are presented in Table 3.

**Figure 6:**
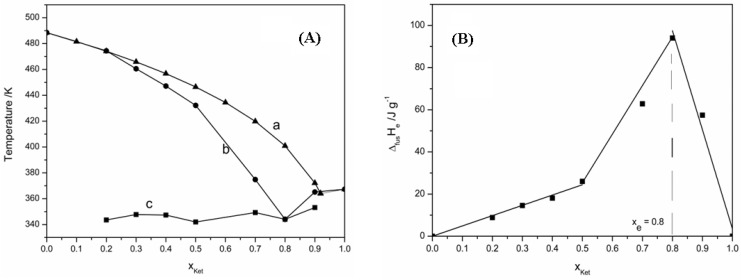
(**A**). Phase diagram of binary mixture Ket—D-HCl: a. ideal behavior; b. real behavior; c. eutectic temperature. (**B**) Tammann’s plot.

By plotting the melting enthalpy based on the molar fraction of Ketoprofen the Tammann diagram was obtained and the eutectic composition can be precisely determined. Tammann’s plot for the binary system Ket—D-HCl is presented in Figure 6B. The phase diagram calculated using the ideal Schroder van Laar equation and the real phase diagram obtained from DSC data is presented in Figure 6A as a temperature-composition plot.

According to the Tammann diagram, the eutectic composition corresponds to a molar fraction of 0.8 Ket. From the SLE phase diagram, it can be seen that up to a composition of the mixture equal to the molar fraction of 0.5 Ket this mixture is characterized by an ideal behavior and for the rest of the molar fractions range the behavior of the system deviates from the ideal one.

Also, for this mixture, it is observed that for the molar fractions range of Ketoprofen 0–0.5 the excess Gibbs free energy value is positive suggesting the existence of weak interactions between the components forming the eutectic melt and for the rest of the molar fraction range, the excess Gibbs free energy value is negative, indicating the existence of weak interactions between molecules of the same type and some stronger among the different molecules, so between Ketoprofen and Drotaverine Hydrochloride.

By calculating the mixing enthalpy using relation (3) for the eutectic composition it follows that this binary system has a cluster structure, the value of the mixing enthalpy being negative and equal to −5.23 kJ mol^−1^.

### 3.3. XRD Analysis

The XRD analysis of pure APIs (Table 4) showed that for Drotaverine Hydrochloride the estimated particle size was 40 nm, for Ibuprofen the estimated particle size was 80 nm, and only 32 nm for Ketoprofen in accord with literature data [21]. The mixtures do not display any additional phases and the lack of appearance of new diffraction peaks indicates the fact that the nanoparticles have not reacted during the mixing process. These results support the conclusion of the DSC study that showed the formation of cluster type Ket—D-HCl and Ibu—D-HCl binary mixtures.

### 3.4. Analysis by FTIR Spectrometry

**Figure 7:**
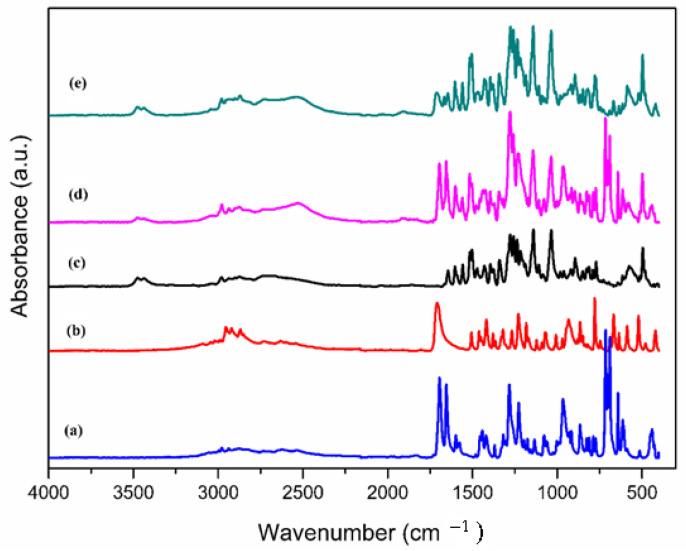
FTIR spectra of (a) Ketoprofen, (b) Ibuprofen, (c) Drotaverine hydrochloride, (d) Ketoprofen- Drotaverine hydrochloride (e) Ibuprofen-Drotaverine hydrochloride.

FTIR spectroscopy is a useful tool in the investigation of molecular interactions appearing between the API components of physical mixtures [22,23,24,25,26]. Pure ibuprofen, ketoprofen, and drotaverine hydrochloride were the subject of several FTIR studies [27,28,29,30,31,32] using different techniques but to the best of our knowledge, their mixtures were never studied before. The FTIR spectra and band assignments obtained for pure APIs and their corresponding eutectic binary mixtures are presented in Figure 7 and Table 5.

Major peaks assignments of Ket, Ibu, and D-HCl of Table 5 agree with the literature data [27,28,29,30,31,32]. As can be seen from Figure 7, the FTIR analysis of the nanosized solid mixtures of Drotaverine Hydrochloride with Ibuprofen, respectively Ketoprofen shows distinctive FTIR peaks at about the same wavenumber values of pure API’s which indicates the absence of chemical transformation in the mixed forms. Ket, Ibu, and D-HCl have only one Hydrogen Bond Donor (HBD) center each and 3, 2, and 5 respectively Hydrogen Bond Acceptor (HBA) centers so Ket is more susceptible to interact by hydrogen bonds with D-HCl than Ibu. According to the Pubchem database Ket has a higher Topological Polar Surface Area (TPSA) of 54.4 Å as compared with that of Ibu of 37.3 Å and thus it is more susceptible to interacting with D-HCl by electrostatic forces [33]. Also, Ket is more hydrophobic i.e., has a lower logP of 3.1 compared to Ibu’s logP of 3.5 [33], being more prone to interact with D-HCl by π-π stacking. As expected, the more hydrophobic character of Ket is due to the presence of a supplementary aromatic cycle. Considering the above structural characteristics: the number of Hydrogen Bond Acceptor centers, the values of Topological Polar Surface Area, and logP results that Ket will develop stronger attractive forces with D-HCl than Ibu. Indeed, larger bands shifts are observed in the FTIR spectra (see please Supplementary Figures S1–S5 data) for Ketoprofen—Drotaverine hydrochloride as compared with Ibuprofen—Drotaverine hydrochloride mixtures in correlation with larger mixing enthalpy obtained from DSC and the above-named structural characteristics. Thus, in the Ibu—D-HCl FTIR spectra are observed: small shifts of about 1 cm^−1^ for the 3475, 1645, 1558, 1503, 1258, 1141 cm^−1^ bands of D-HCl, of about 2 cm^−1^ for the 3437 and 1708 cm^−1^ bands of D-HCl respectively Ibu, of about 4 cm^−1^ for the 2877 cm^−1^ band of D-HCl and of 5 cm^−1^ for the 1418 cm^−1^ band of Ibu. In the Ket—D-HCl spectra slightly larger shifts are observed as follows: of about 2 cm^−1^ for 1558, 1258, 1141, and 1694 cm^−1^ bands of D-HCl respectively Ket, of about 3 cm^−1^ for the 3437 and 1598 cm^−1^ bands of D-HCl respectively Ket, of about 4 cm^−1^ for the 2901 and 1472 cm^−1^ bands of D-HCl and about 6 cm^−1^ for the 1645 cm^−1^ band of D-HCl. Band shifting is observed principally for O-H, C-Harom, C=C, and C=O modes of pure APIs demonstrating the presence of intermolecular interactions by hydrogen bonds, π-π stacking, and general van der Waals interaction forces.

## 4. Conclusions

Mechanosynthesis, a simple, rapid, and ecological method was used for the preparation of the binary systems of APIs. The DSC measurements were carried out for the pre-, post, and eutectic mixtures of APIs and highlighted the compatibility between components. The XRD and FTIR measurements showed that the binary mixtures are formed by weak physical forces and a chemical reaction did not took place during their preparation. The formation of a cluster-type eutectic mixture in the process of mixing the powders of active principles is the most probable. The two binary systems: Ibuprofen—Drotaverine hydrochloride and Ketoprofen—Drotaverine hydrochloride show a simple eutectic behavior. This can be seen also in the SLE phase diagrams along with the deviation from the ideal behavior of the mixtures. The negative value of enthalpy of mixing for the binary systems suggests the formation of clustering of molecules in the binary eutectic melt. From a physicochemical point of view, this study highlights the potential use of a eutectic mixture of Ibu, Ket, and D-HCl below the eutectic temperature. The negative values of excess thermodynamic function *G^E^* for the binary eutectic mixtures of Drotaverine Hydrochloride–Ibuprofen and Drotaverine Hydrochloride—Ketoprofen showed the presence of strong interactions between different molecules with the formation of clusters of molecules (the negative value of the mixing enthalpy).

The eutectics obtained can be a viable option for the treatment of pain associated with cancer therapy. One way to test this claim is to study these eutectic mixtures on several human tumor cell lines.

## Figures and Tables

**Table 1:** Melting temperatures and enthalpy of fusion of APIs *.

Pure Compound	*T_fus_*/K		∆*_fus_H*/kJ mol^−1^
	Lit. (DSC)	This Study (DSC)	This Study (DSC)
Ibuprofen	349 [19]	348.4 ± 0.2	24.91 ± 0.43
Ketoprofen	366.15 [18]	367.5 ± 0.2	27.42 ± 0.32
Drotaverine Hydrochloride	489.35 [20]	488.4 ± 0.4	50.52 ± 0.52

*—Results were statistically significant with *p*-values smaller than 0.05.

**Table 2:** Thermodynamic parameters for Drotaverine Hydrochloride and Ibuprofen system *.

x_Ibu_	1st DSC Peak		2nd DSC Peak	*G^E^*/J mol^−1^
	*T*/K	Δ*_fus_H_e_*/J g^−1^	*T*/K	
0	-	-	488.4	-
0.2	343.3	14.77	474.2	2900.5
0.4	343.8	31.54	457.6	4065.4
0.5	343.5	51.68	443.3	3987.2
0.7	343.5	75.48	412.8	3175.2
0.9	343.8	111.9	343.8	−615.4
1	-	-	348.4	-

*—Results were statistically significant with *p*-values smaller than 0.05.

**Table 3:** Thermodynamic parameters for Drotaverine Hydrochloride and Ketoprofen system *.

x_Ket_	1st DSC Peak		2nd DSC Peak	*G^E^*/J mol^−1^
	*T*/K	∆*_fus_H_e_*/J g^−1^	*T*/K	
0	-	-	488.4	-
0.2	343.6	8.86	474.4	2582.4
0.3	347.8	14.56	460.5	2553.5
0.4	347.4	18.09	447.2	2450.0
0.5	342.0	26.03	432.2	2102.5
0.7	349.3	62.82	374.8	−1217.7
0.8	344.1	94.02	344.1	−1828.0
0.9	353.2	57.44	365.2	−470.1
1	-	-	367.4	-

*—Results were statistically significant with *p*-values smaller than 0.05.

**Table 4:** Main XRD relative intensities for nanoparticles single components and mixtures *.

Ket		Ibu		D-HCl	
*Angle (2* *ϴ* *)*	*I/I_0_*	*Angle (2* *ϴ* *)*	*I/I_0_*	*Angle (2* *ϴ* *)*	*I/I_0_*
23	100	6	100	14.5	100
18.5	79.3	22.3	81.4	22	60
22	69.4	16	65.4	44	28.57
6.5	46.2	20.4	52.7	65	25.42
19.5	51.3	12.3	30.7	77	27.71

*—Results were statistically significant with *p*-values smaller than 0.05.

**Table 5:** FTIR absorption peaks for Ibu, D-HCl, and Ket and band assignments (v-stretching, δ-deformation).

Ibu		D-HCl		Ket	
*ν* (cm^−1^)	*Vibration Mode*	*ν* (cm^−1^)	*Vibration Mode*	*ν* (cm^−1^)	*Vibration Mode*
2955	νCH_3_ antisymmetric	3500–3300	νN-H secondary amine	3054	νC-H
1708	νC=O	3000–2840	νC-H		
1507	νC=C aromatic	1650–1580	δ N-H	1694	νC=O
1418	δCH-CO	1600–1475	νC=C aromatic	1654	νC=O (ketone)
1329	δOH in plane	1260–1000	νC-O	15,898	νC=C aromatic
1230	νC-C			1442	νC=C aromatic
934	δCH_3_ rocking				
866	δC-H out of plane				
779	δCH_2_ rocking				
668	δC-H out of plane				

## Data Availability

The data supporting reported results are available on request from the authors.

## Supplementary Materials

The following supporting information can be downloaded at: https://www.mdpi.com/article/10.3390/ma15031244/s1, Figure S1: FTIR spectra of ketoprofen; Figure S2: FTIR spectra of ibuprofen; Figure S3: FTIR spectra of drotaverine hydrochloride; Figure S4: FTIR spectra of ketoprofen-drotaverine hydrochloride; Figure S5: FTIR spectra of ibuprofen-drotaverine hydrochloride.
