# Supplementary material for: Compatibility of Drotaverine Hydrochloride with Ibuprofen and Ketoprofen Nonsteroidal Anti-Inflammatory Drugs Mixtures
Source: Materials (Basel). 2022 Feb 8;15(3):1244. doi: 10.3390/ma15031244 (PMC8838396; doi:10.3390/ma15031244)
Supplement: Supplementary file 1 [file materials-15-01244-s001.zip › materials-1553037-supplementary.pdf]

Supplementary Material

Compatibility of Drotaverine Hydrochloride with Ibuprofen and Ketoprofen Nonsteroidal Anti-inflammatory Drugs Mixtures

Andreia-Cristina Soare, Viorica Meltzer, Claudiu Colbea, Ioana Stanculescu and Elena Pincu *

Department of Physical Chemistry, Faculty of Chemistry, University of Bucharest , Bd. Regina Elisabeta 4-12, 030018 Bucharest, Romania; andreia-cristina.soare@drd.unibuc.ro (A.-C.S.); meltzerviorica@yahoo.com (V.M.); ccolbea@ethz.ch (C.C.); ioana.stanculescu@chimie.unibuc.ro (I.S.)

* Correspondence: elena.pincu@chimie.unibuc.ro

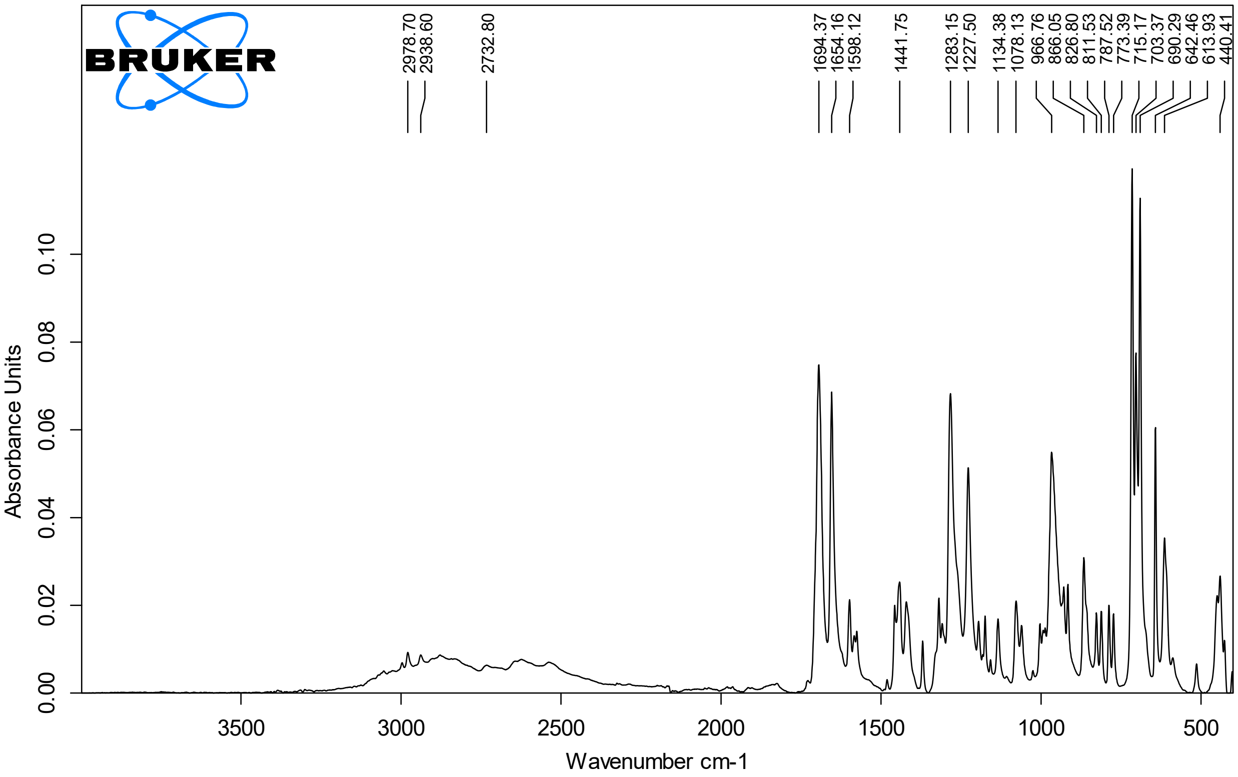

Figure S1. FTIR spectra of ketoprofen.

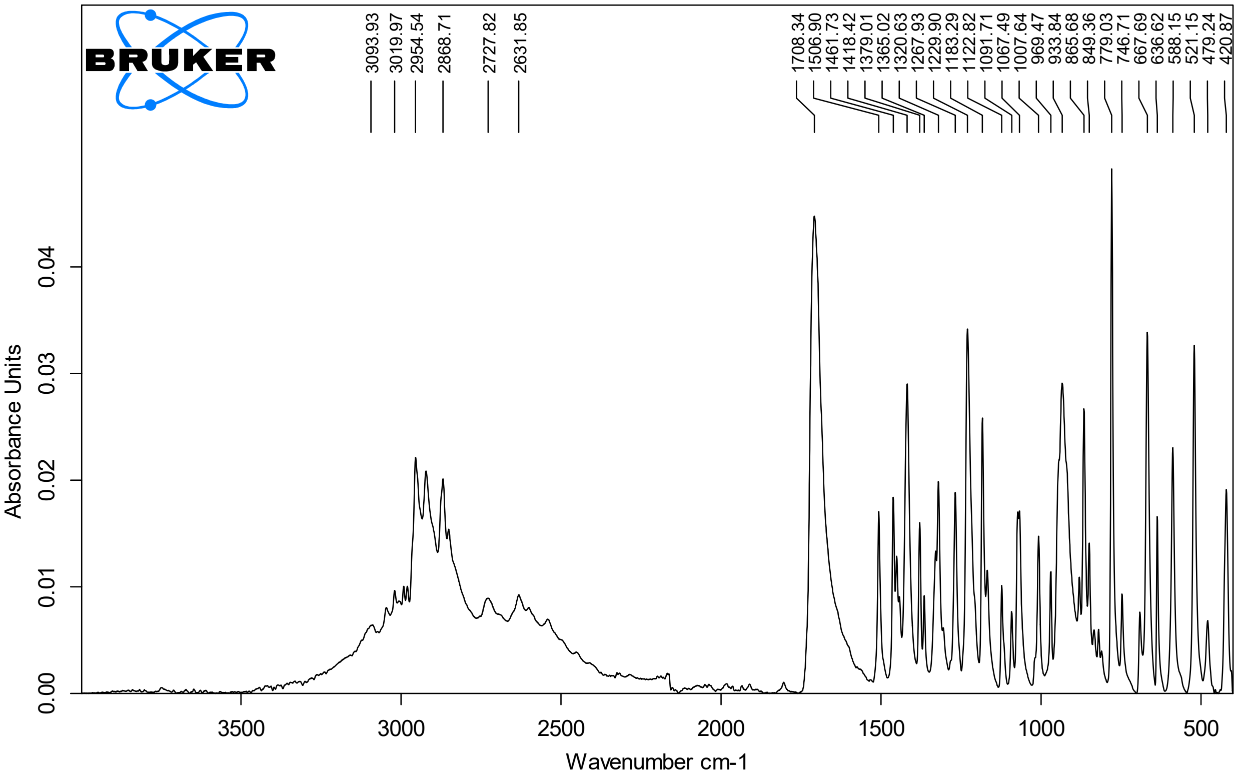

Figure S2. FTIR spectra of ibuprofen.

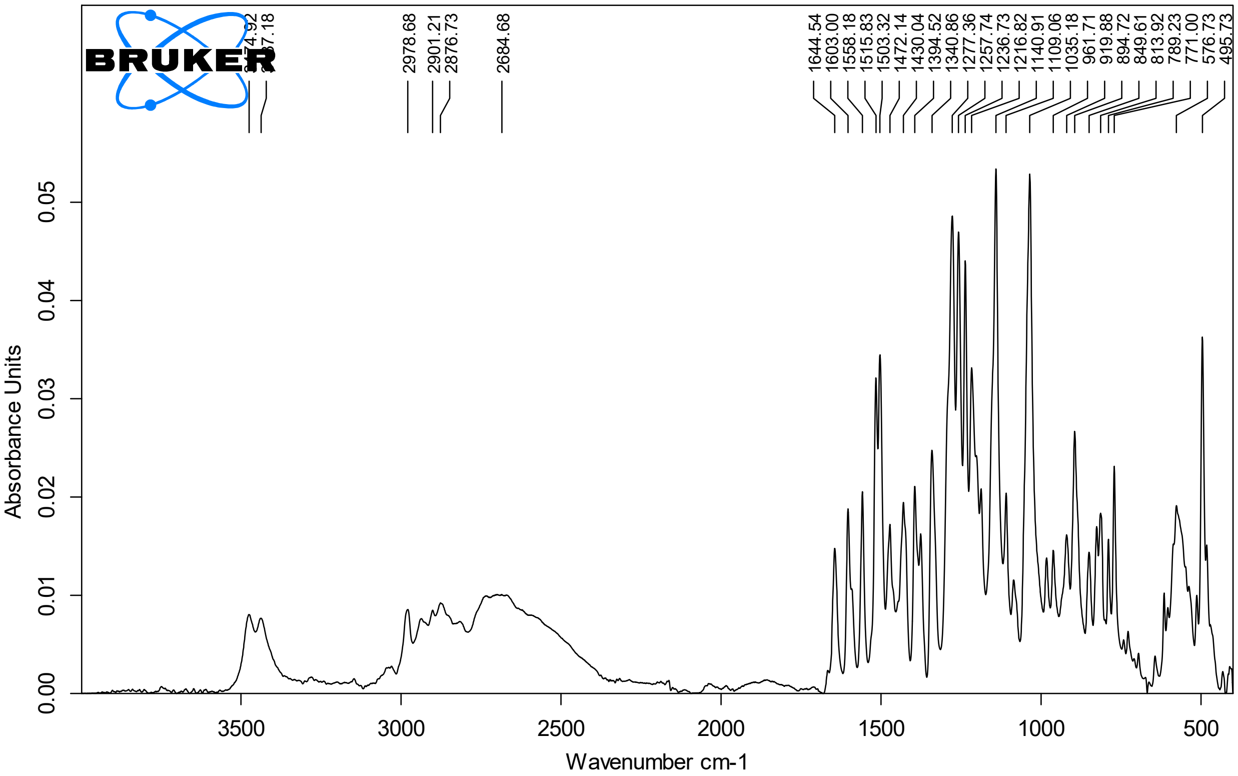

Figure S3. FTIR spectra of drotaverine hydrochloride.

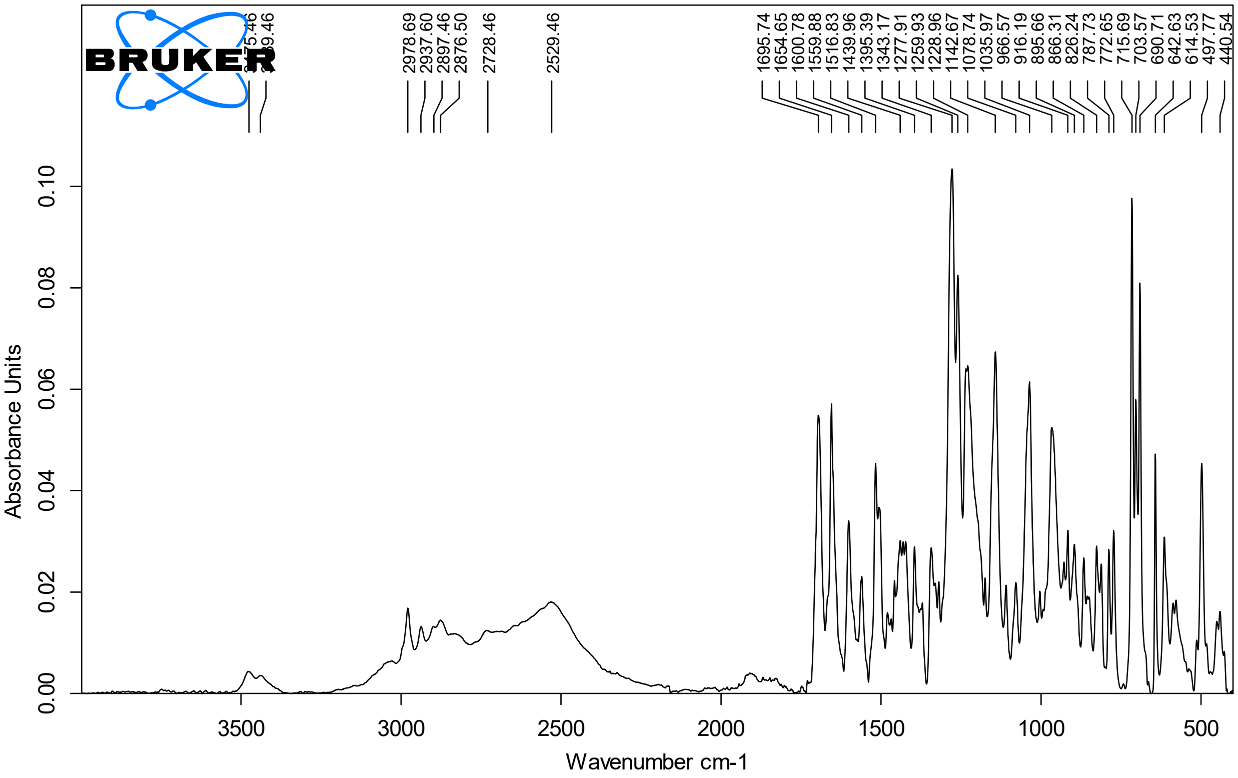

Figure S4. FTIR spectra of ketoprofen-drotaverine hydrochloride.

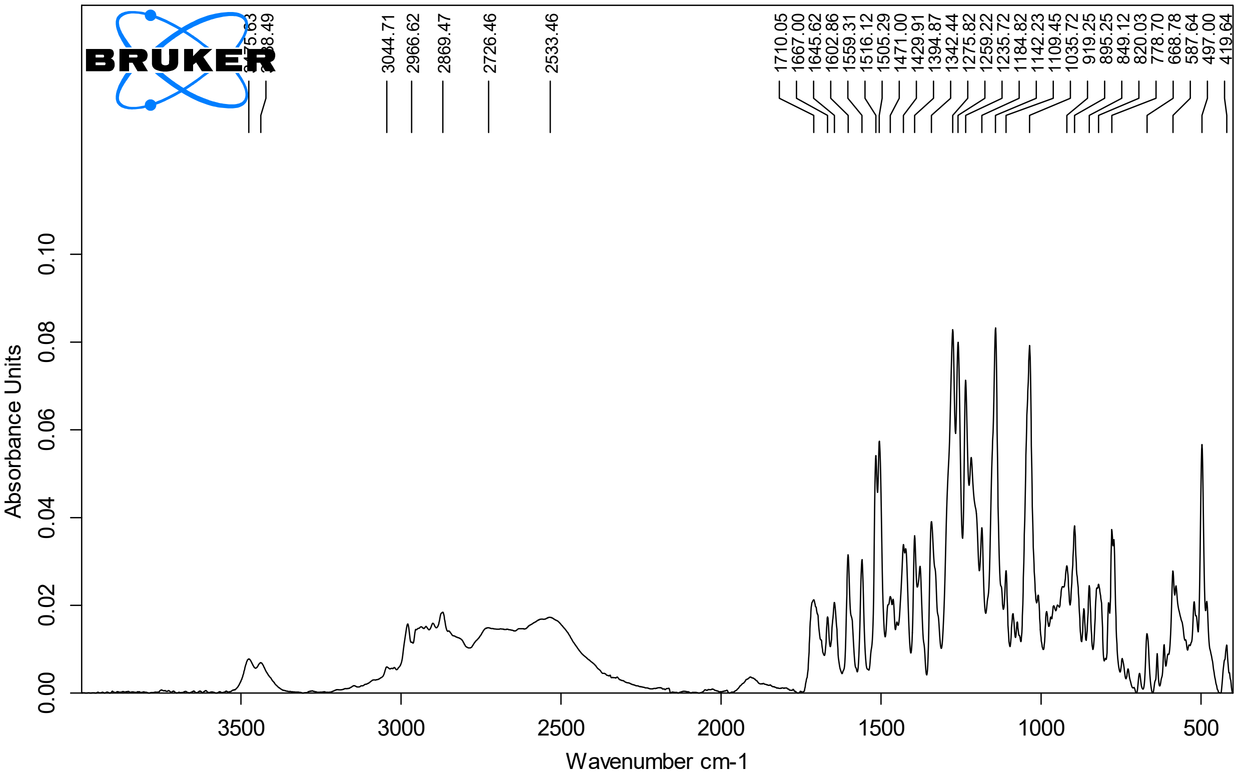

Figure S5. FTIR spectra of ibuprofen-drotaverine hydrochloride.
